# Performance of ChatGPT, Claude, and AMBOSS on the European Board of Urology In-Service Assessment and Alignment With the European Association of Urology 2025 Guidelines: Comparative Study

**DOI:** 10.2196/100148

**Published:** 2026-09-02

**Authors:** Mohamed Eldaneen, Shaza Hendy, Omar Ramadan, Panagiotis Nikolinakos, Asif Muneer, Hussain M Alnajjar, Karl H Pang

**Affiliations:** 1 Department of Urology, Chelsea and Westminster Hospital NHS Foundation Trust London United Kingdom; 2 East Kent Hospitals University NHS Foundation Trust Kent United Kingdom; 3 Division of Surgery and Interventional Science University College London London, WC1E 6BT United Kingdom; 4 University College London Hospitals NHS Foundation Trust London, England United Kingdom

**Keywords:** European Board of Urology, EBU, artificial intelligence, AI, ChatGPT, Claude, AMBOSS, European Association of Urology guidelines, EAU guidelines

## Abstract

**Background:**

Recent advances in AI, particularly large language models, have generated growing interest in their application to medical education and examination preparation. However, the accuracy, reasoning quality, and adherence to clinical guidelines of these tools in postgraduate urology assessments remain unclear.

**Objective:**

This study aimed to evaluate the performance of 3 AI tools, ChatGPT (GPT-4.0), Claude (version 4.5), and AMBOSS, on European Board of Urology (EBU)–style multiple-choice questions, with a particular focus on accuracy, insight, concordance, and adherence to European Association of Urology (EAU) guidelines.

**Methods:**

A total of 200 single-best-answer questions from the EBU In-Service Assessment workbook (2021-2022) were input into each AI model. Models were prompted to select an answer and provide an explanation. Two urologists with post–Fellowship of the Royal College of Surgeons (FRCS) training independently assessed the outputs. Accuracy was defined as correct answer selection. Concordance was defined as the logical alignment between the answer and its explanation. Insight was evaluated across 3 domains—nonobvious deduction, discriminative reasoning, and clinical validity—and was graded as low, moderate, or high.

**Results:**

ChatGPT demonstrated the highest accuracy (171/200, 85.5%), compared to Claude and AMBOSS (both 159/200, 79.5%; *P*=.14). Concordance was also significantly higher for ChatGPT (190/200, 95%) than for Claude (176/200, 88%) and AMBOSS (152/200, 76%; *P*<.001). Nonobvious deduction was predominantly low to moderate across all models, reflecting the recall-based nature of many questions. ChatGPT and Claude showed stronger discriminative reasoning, while AMBOSS demonstrated limited exclusion of alternative options. Clinical validity was high overall, with ChatGPT showing the greatest consistency with EAU guidelines. There was substantial agreement between the 2 reviewers (weighted κ coefficient >0.61).

**Conclusions:**

AI tools can achieve high accuracy on EBU-style assessments; however, differences in reasoning quality and guideline adherence are evident. ChatGPT demonstrated superior performance across all evaluated domains, supporting its role as a potential adjunct in postgraduate urology education.

## Introduction

The European Board of Urology (EBU) examination is a high-stakes assessment designed to evaluate core and advanced urological knowledge in trainees approaching completion of specialist training. Success in the EBU examination is often viewed as an indicator of readiness for independent practice and is closely aligned with the knowledge base required for fellowship-level examinations, such as the Fellowship of the Royal College of Surgeons (FRCS) exam [1].

The EBU examination is a 2-part assessment comprising a written theory examination (part 1) and an oral viva examination (part 2). The part 1 written examination consists of 110 single-best-answer multiple-choice questions (MCQs) designed to determine whether candidates meet the minimum knowledge standard defined by the EBU. The examination covers the breadth of urological practice and is structured across core domains, including basic science, oncology, endourology, andrology, functional urology, trauma, and other subspecialty areas. The part 2 examination is an oral viva that assesses clinical reasoning and decision-making through structured case-based discussions [1].

Advances in AI, particularly the development of large language models (LLMs), have generated substantial interest in medical education [2,3]. Despite this promise, the application of LLMs in health care education remains contentious due to concerns regarding factual accuracy, hallucinated outputs, lack of transparency in reasoning, and the risk of overreliance by learners [4]. Although several studies have demonstrated strong LLM performance in general medical and nonmedical professional examinations, including the United States Medical Licensing Examination (USMLE) and legal board assessments [5-7], performance within specialty-specific, higher-order clinical domains, particularly those requiring nuanced decision-making, remains less well characterized [8]. Recent work has suggested that AI systems enhanced with specialty-specific guidelines can achieve high performance on urology board-style questions, highlighting both the potential and limitations of such tools in specialist education [9].

In this study, we aimed to evaluate the performance of 3 widely used AI tools: 2 general-purpose models, ChatGPT (GPT-4.0) and Claude (version 4.5), and 1 medical-specific platform, AMBOSS, on a set of EBU-style urology MCQs. Unlike general LLMs, AMBOSS’s content and explanations are curated by medical educators and clinicians, which may enhance factual accuracy and guideline alignment in domain-specific settings [10].

In addition, model outputs were assessed for insight and concordance with expert reasoning, using benchmark comparisons against the European Association of Urology (EAU) guidelines [11].

## Methods

This study was performed with reference to the METRICS (Modern Quality Assessment Framework for Evaluating Generative AI Studies in Healthcare) framework [12] (Multimedia Appendix 1).

### Study Design

This was a comparative evaluation of 3 AI-based tools, ChatGPT (GPT-4.0; OpenAI), Claude (version 4.5; Anthropic PBC), and AMBOSS (AMBOSS GmbH), on EBU-style MCQs, benchmarked against expert clinician assessment using the 2025 EAU guidelines [8] as a reference.

Data collection was performed between December 8 and 12, 2025. ChatGPT was accessed using a ChatGPT Plus subscription through the web interface [13], Claude through the Claude-4.5 Pro web interface [14], and AMBOSS through a full-access institutional subscription [15].

Questions were entered manually using an identical prompt for all models: “You are sitting the European Board of Urology (EBU) written examination. For the following question, select the single best answer from the options provided. Provide your chosen answer and a brief explanation for your reasoning.”

Each question was submitted once in a new, empty conversation to minimize context carryover between responses. No system prompts, custom instructions, or user-modifiable parameters were used. Output variability across repeated runs was not assessed and represents a limitation of the study.

### Question Source

The most recent available EBU In-Service Assessment Questions booklet (2021-2022) was requested from the EBU by email, and approval was obtained for its use. This official workbook contains 200 single-best-answer MCQs with predetermined correct answers. The In-Service Assessment and its accompanying workbook are not official past papers of the EBU part 1 written examination but serve as structured preparatory and educational resources reflecting the style and scope of the certification examination and do not operate on a pass or fail basis.

EBU examination outcomes are reportedly based on the mean score and SD of the candidate cohort, although official pass rates are not publicly available. Furthermore, the workbook is an educational resource. Therefore, performance in this study cannot be directly equated with performance on the official EBU part 1 examination.

### Assessment

Accuracy was defined as whether the selected answer correctly addressed the question (correct vs incorrect).

Concordance was defined as the logical alignment between the selected answer and its accompanying explanation, consistent with previously described LLM evaluation frameworks. Concordance was assessed on a per-question basis, with each of the 200 questions scored as either “yes” (in agreement with the expected answer) or “no.” The concordance percentage was then calculated.

Insight assessment was adapted from prior studies evaluating the presence of nonobvious, clinically meaningful reasoning in AI-generated responses and was assessed across three domains: (1) nonobvious deduction (reasoning beyond the question stem), (2) discriminative reasoning (active exclusion of alternative options), and (3) clinical validity (factual accuracy and alignment with accepted urological practice and the EAU guidelines). Insight was graded as low (minimal or absent reasoning), moderate (partial reasoning), or high (clear, structured, and multistep reasoning). A 3-tier classification was selected to capture gradations in reasoning quality and explanatory depth that may not be adequately represented by a binary assessment [16,17].

Ratings were independently assigned by 2 assessors (2 post-FRCS urologists, ME and OR), and disagreements were resolved through consensus discussion, with adjudication by a senior reviewer (KHP) where required, consistent with established approaches for expert-rated assessment of complex constructs [18].

### Analysis

Descriptive data were presented as numbers and percentages with 95% CI, where appropriate. Paired categorical data were analyzed using the McNemar test for comparisons between 2 related groups and the Cochran *Q* test for comparisons among 3 or more related groups. Statistical significance was defined as *P*<.05. The weighted κ coefficient was used to assess interrater reliability for agreement on concordance and insight categorical data [19]. Results were tabulated, and graphs were plotted using Microsoft Excel (version 16).

## Results

The accuracy and concordance performance of the 3 AI models with corresponding 95% CIs are summarized in Table 1. ChatGPT achieved the highest accuracy at 85.5% (171/200, 95% CI 80.6%-90.4%), compared with 79.5% (159/200, 95% CI 73.9%-85.1%) for both Claude and AMBOSS (*P*=.14). All 3 models achieved accuracy scores exceeding 70%, indicating a high level of performance on EBU-style questions.

**Table 1:** Accuracy and concordance of ChatGPT, Claude, and AMBOSS, with 95% CIs (N=200).

Outcomes and models			Positive responses, n (%; 95% CI)		Cochran Q		*P* value
**Accuracy**					3.9		.14^a^
	ChatGPT	171 (85.5; 80.6-90.4)					
	Claude	159 (79.5; 73.9-85.1)					
	AMBOSS	159 (79.5; 73.9-85.1)					
**Concordance**					29.3		*<.001* ^a,b^
	ChatGPT	190 (95; 92.0-98.0)				*.02* ^c^	
	Claude	176 (88; 83.5-92.5)				*<.001* ^b,d^	
	AMBOSS	152 (76; 70.1-81.9)				*.004* ^b,e^	

^a^Comparison between the 3 AI tools (Cochran Q test).

^b^Statistically significant (*P*<.05).

^c^Comparison between ChatGPT and Claude (McNemar).

^d^Comparison between ChatGPT and AMBOSS.

^e^Comparison between Claude and AMBOSS.

ChatGPT significantly demonstrated the highest concordance at 95% (190/200, 95% CI 92.0%-98.0%), followed by Claude at 88% (176/200, 95% CI 83.5%-92.5%) and AMBOSS at 76% (152/200, 95% CI 70.1%-81.9%; *P*<.001). There were also statistically significant differences observed between the different AI tools (Table 1). The weighted κ coefficient was 1.0, representing perfect agreement.

When analyzing subspecialty topics, all 3 models achieved an accuracy of at least 68.1% across all domains (Table 2). An accuracy of 100% was achieved by all models in “miscellaneous” and “transplant and nephrology.” ChatGPT and Claude both achieved 100% accuracy in “trauma or emergency.” ChatGPT achieved the highest accuracy in “oncology” (82.6%), “pediatric and congenital urology” (90.9%), and “surgical principles” (100%). Claude performed best in “functional urology and benign prostatic hyperplasia” (85.7%) and “lithiasis and infections” (84.6%), while AMBOSS achieved the highest accuracy in “andrology and infertility” (93.8%).

**Table 2:** Accuracy of ChatGPT, Claude, and AMBOSS across European Board of Urology subspecialty domains.

Domains	ChatGPT, accuracy (%)	Claude, accuracy (%)	AMBOSS, accuracy (%)
Andrology and infertility	81.3	81.3	93.8
Functional urology and benign prostatic hyperplasia	75.0	85.7	75.0
Lithiasis and infections	80.8	84.6	80.8
Miscellaneous	100	100	100
Oncology	82.6	68.1	76.8
Pediatric and congenital urology	90.9	81.8	68.2
Surgical principles	100	86.7	80.0
Transplant and nephrology	100	100	100
Trauma or emergency	100	100	75.0

**Figure 1:**
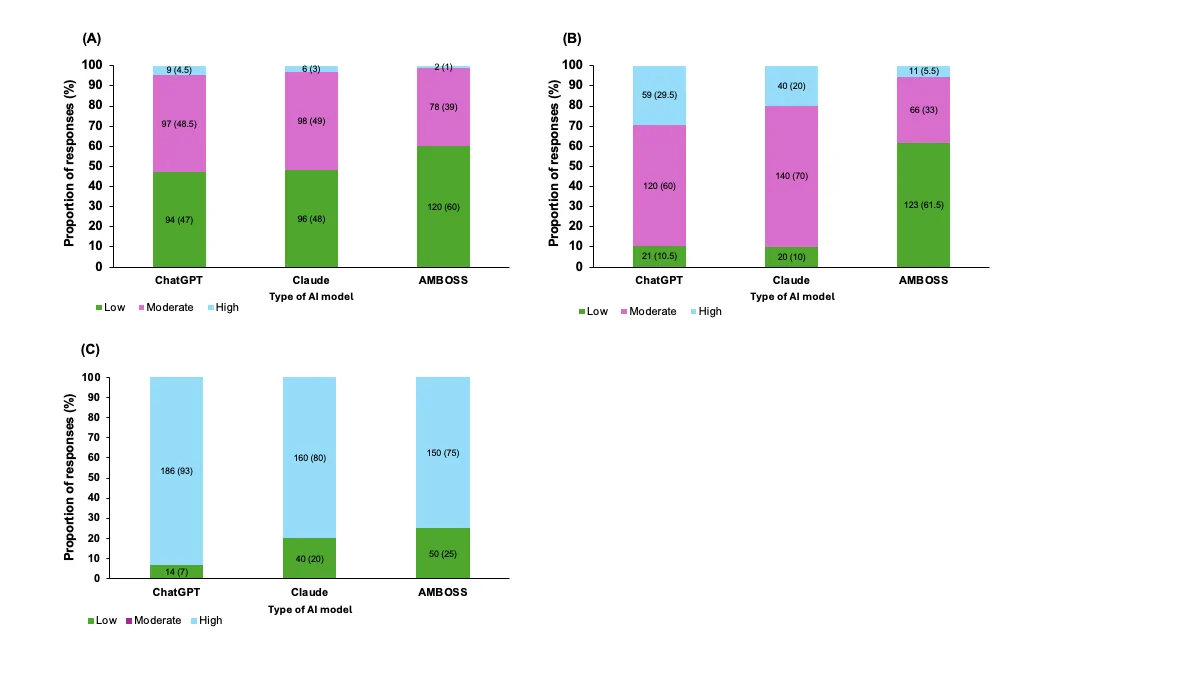
Insight assessment across AI models. (A) Nonobvious deduction, (B) discriminative reasoning, and (C) clinical validity. Bars represent the percentage of 200 questions rated as low, moderate, or high insight for ChatGPT, Claude, and AMBOSS.

The results from the insight assessment are demonstrated in Figure 1 and Multimedia Appendix 2. For nonobvious deduction, ChatGPT demonstrated low, moderate, and high ratings in 47% (94/200), 48.5% (97/200), and 4.5% (9/200) of responses, respectively. Claude showed a similar distribution, with low, moderate, and high ratings in 48% (96/200), 49% (98/200), and 3% (6/200) of responses, respectively. AMBOSS demonstrated the lowest performance in this domain, with low, moderate, and high ratings in 60% (120/200), 39% (78/200), and 1% (2/200) of responses, respectively (Figure 1A).

For discriminative reasoning, ChatGPT achieved low, moderate, and high ratings in 10.5% (21/200), 60% (120/200), and 29.5% (59/200) of responses, respectively. Claude demonstrated low, moderate, and high ratings in 10% (20/200), 70% (140/200), and 20% (40/200) of responses, respectively. AMBOSS achieved low, moderate, and high ratings in 61.5% (123/200), 33% (66/200), and 5.5% (11/200) of responses, respectively (Figure 1B).

Clinical validity was highest for ChatGPT, with 93% (186/200) of responses rated as high and 7% (14/200) rated as low. Claude achieved high and low ratings in 80% (160/200) and 20% (40/200) of responses, respectively, while AMBOSS achieved high and low ratings in 75% (150/200) and 25% (50/200) of responses, respectively (Figure 1C).

Substantial agreement was observed between the 2 reviewers. The weighted κ coefficient was 0.67, 0.73, and 0.80 for nonobvious deduction, discriminative reasoning, and clinical validity, respectively.

## Discussion

### Principal Findings

In this study, we evaluated the performance of ChatGPT, Claude, and AMBOSS on 200 EBU-style MCQs using a multidimensional assessment framework that examined not only answer accuracy but also concordance, nonobvious deduction, discriminative reasoning, and clinical validity. Although previous studies have demonstrated that LLMs can achieve pass-level or near–pass-level performance on medical and specialty examinations, these evaluations have largely focused on answer correctness and examination outcomes [20-23]. In contrast, our study assessed the quality and transparency of the reasoning underpinning AI-generated responses, with a particular emphasis on adherence to EAU guideline–based practice.

Previous work supports the growing role of AI in medical education, with GPT-based models demonstrating strong performance across licensing and board examinations [20-23].

However, most studies have focused primarily on examination accuracy rather than the reasoning processes underlying responses. For example, Vaishya et al [24] evaluated AI performance on orthopedic postgraduate examination questions without systematically assessing explanation quality. Similarly, studies of medical question-answering systems and patient-facing chatbots have shown encouraging performance but have also highlighted that answer correctness alone may not adequately capture response quality, transparency, safety, or clinical applicability [25,26].

Assessing guideline adherence is particularly important given the known limitations of AI-generated clinical recommendations. Talyshinskii et al [27] found that although GPT-4.0 demonstrated partially accurate diagnostic knowledge in urolithiasis, its surgical planning recommendations were not consistently aligned with EAU guidelines. Additionally, Eldaneen et al [28] reported that current LLMs provide readily accessible guidance on LUTS; however, their unsupervised use in clinical decision-making remains a concern and may be considered premature.

Broader evaluations of generative AI in health care have similarly highlighted concerns regarding reliability, hallucinations, safety, and the need for human oversight [29,30]. Furthermore, Topol [31] argued that successful integration of AI into health care depends on maintaining human clinical oversight, while systematic reviews have identified ongoing concerns regarding reliability, interpretability, and real-world applicability [32]. Together, these findings emphasize the importance of evaluating not only whether an answer is correct but also whether the underlying reasoning is transparent and clinically appropriate.

Although all 3 models achieved an accuracy exceeding 70%, important differences emerged when reasoning quality was assessed directly. ChatGPT demonstrated the strongest overall performance, with the highest accuracy, concordance, insight scores, and clinical validity. This finding is consistent with recent reports showing strong performance of GPT-4.0–based models across medical knowledge and clinical reasoning assessments [33]. Although all models achieved scores exceeding 70% on the EBU In-Service Assessment workbook, these results should not be interpreted as equivalent to performance on the official EBU part 1 examination because the workbook is an educational resource rather than a validated examination used for certification.

ChatGPT more frequently incorporated structured, multistep reasoning; guideline-based interpretation; and explicit exclusion of alternative answers, resulting in the highest concordance scores.

Claude achieved comparable accuracy and demonstrated strong clinical validity and discriminative reasoning. Its explanations frequently justified why alternative answers were incorrect and were generally consistent with accepted urological practice. However, compared with ChatGPT, Claude was less likely to generate reasoning that extended beyond the information explicitly provided in the question stem.

AMBOSS also demonstrated good overall accuracy and guideline alignment, but its explanations were typically shorter and more descriptive. Although correct answers were often provided, the explanations were less likely to justify the selected option or actively exclude competing alternatives. This was reflected in lower concordance and insight scores than those of ChatGPT and Claude.

High levels of nonobvious deduction were uncommon across all models. This likely reflects the structure of EBU-style questions, many of which assess factual recall and therefore provide limited opportunities for advanced reasoning. Higher ratings were generally observed in questions requiring the integration of multiple clinical concepts or the application of guideline-based management decisions.

The greatest differences between models were observed in discriminative reasoning. ChatGPT and Claude more frequently compared competing answer options and explicitly explained why incorrect alternatives should be excluded. In contrast, AMBOSS often identified the correct answer without fully articulating the reasoning process that led to that conclusion. The ability to actively exclude alternative options represents an important component of both examination performance and clinical decision-making and may enhance the educational value of AI-generated explanations.

All 3 models demonstrated strong clinical validity overall, with most responses aligning with accepted urological practice and EAU guideline recommendations. ChatGPT demonstrated the highest consistency in this domain. These findings are reassuring from an educational perspective, suggesting that clinically inappropriate or potentially misleading recommendations were relatively uncommon across all 3 platforms.

Our findings provide further insight into previously reported limitations of AI performance in medical assessments. Although studies such as those by Sadeq et al [34] have demonstrated variability in chatbot performance across question types and levels of complexity, our results suggest that differences between models are not fully explained by accuracy alone. Models with similar examination performance may differ substantially in how they justify their answers, exclude alternative options, and maintain alignment with guideline-based practice. This observation is consistent with the work of Holzinger et al [35], who emphasized the importance of evaluating explanations rather than outputs alone.

On the basis of the findings of this study, AMBOSS may be particularly useful for factual revision, whereas ChatGPT and Claude may offer additional value for examination preparation because of their more detailed reasoning processes. Nevertheless, none of the evaluated models should be considered substitutes for clinical supervision or contemporary guideline consultation.

Overall, AI should be introduced in a staged and supervised manner, supporting rather than replacing traditional educational approaches, consistent with recent recommendations regarding the integration of LLMs into medical education [36,37].

### Limitations

Several limitations should be acknowledged. This study was limited to single-best-answer MCQs and did not assess performance in more complex or multimodal scenarios, such as imaging interpretation, operative planning, or real-world clinical decision-making. The EBU In-Service Assessment workbook was used as an educational benchmark and is not equivalent to the official EBU part 1 examination; therefore, the findings should not be interpreted as reflecting performance on the certification examination itself. Although predefined criteria, assessor calibration, and dual independent raters were used, some subjectivity is unavoidable. In addition, AI models are dynamic systems whose outputs may change over time because of model updates, and responses were evaluated at a single time point without assessment of repeated-run variability or reproducibility. Potential overlap between model training data and educational resources used in this study cannot be excluded. Furthermore, AI models remain dependent on the scope and currency of their training data, raising concerns regarding alignment with evolving clinical guidelines. Finally, performance in a controlled examination setting may not reflect real-world clinical use.

### Conclusions

AI tools demonstrate strong performance on postgraduate urology examination-style questions but differ in reasoning quality and educational value. ChatGPT showed the highest combined accuracy, concordance, and insight, with consistent guideline adherence and strong discriminative reasoning. Claude demonstrated comparable accuracy with good clinical validity but less consistent deductive depth. AMBOSS provided accurate, guideline-aligned responses but relied more on descriptive knowledge with limited explicit reasoning. These findings support the structured and critical use of AI as an adjunct in urology education, emphasizing tools that promote transparent, guideline-based clinical reasoning.

## Data Availability

All data generated or analyzed during this study are included in this published article.

## Appendix

Multimedia Appendix 1 Compliance with the METRICS (Modern Quality Assessment Framework for Evaluating Generative AI Studies in Healthcare) checklist.

Multimedia Appendix 2 Nonobvious deduction, discriminative reasoning, and clinical validity insight levels across AI models.

## Abbreviations

EAU: European Association of Urology
EBU: European Board of Urology
FRCS: Fellowship of the Royal College of Surgeons
LLM: large language model
MCQ: multiple-choice question
METRICS: Modern Quality Assessment Framework for Evaluating Generative AI Studies in Healthcare
USMLE: United States Medical Licensing Examination
