# Supplementary material for: Performance of ChatGPT, Claude, and AMBOSS on the European Board of Urology In-Service Assessment and Alignment With the European Association of Urology 2025 Guidelines: Comparative Study
Source: JMIR Form Res. 2026 Sep 2;10:e100148. doi: 10.2196/100148 (PMC13583496; doi:10.2196/100148)
Supplement: Supplementary file 1 [file formative_v10i1e100148_app1.docx]

**Multimedia Appendix 1.** METRICS Checklist Compliance.

This checklist was completed using the METRICS framework for reporting and evaluation of generative artificial intelligence studies in healthcare. The checklist demonstrates transparency regarding model specification, prompt design, evaluation methodology, statistical reporting, reproducibility considerations, and study limitations.

| **METRICS Domain** | **Compliance** | **Evidence from Current Study** | **Location** |
| --- | --- | --- | --- |
| Model transparency | Yes | ChatGPT-4 Plus, Claude 4.5 Pro, and AMBOSS identified together with access platform. | Methods |
| Model access details | Yes | Web-based interfaces used; subscriptions and institutional access reported. | Methods |
| Evaluation objective | Yes | Comparison of AI performance on EBU-style MCQs against expert assessment and EAU guidelines. | Introduction/Methods |
| Dataset description | Yes | 200 MCQs from the EBU In-Service Assessment Workbook 2021–2022. | Methods |
| Input specification | Yes | Identical prompt used across all models. | Methods |
| Prompt transparency | Yes | Full prompt reproduced verbatim. | Methods |
| Prompt standardisation | Yes | Same prompt and workflow applied to all models. | Methods |
| Execution protocol | Yes | Each question submitted once in a new conversation. | Methods |
| Output collection | Yes | Answer selection and explanation recorded for each model. | Methods |
| Human evaluation | Yes | Two post-FRCS urologists independently assessed outputs. | Methods |
| Evaluator expertise | Yes | Assessors were post-FRCS urologists; senior FEBU reviewer adjudicated disagreements. | Methods |
| Assessment framework | Yes | Accuracy, concordance, non-obvious deduction, discriminative reasoning, and clinical validity defined. | Methods |
| Calibration procedure | Yes | Assessors calibrated grading before formal evaluation. | Methods |
| Reproducibility | Partial | Prompt, data source and workflow reported; repeated-run reliability not assessed. | Methods/Limitations |
| Statistical reporting | Yes | Proportions and 95% confidence intervals reported. | Methods/Results |
| Uncertainty reporting | Yes | 95% confidence intervals provided for accuracy and concordance. | Results |
| Bias and limitations | Yes | Potential training-data overlap, lack of repeated-run testing, and absence of Cohen's kappa acknowledged. | Limitations |
| Clinical relevance | Yes | Outputs benchmarked against EAU 2025 guideline recommendations. | Methods/Discussion |
| Transparency of findings | Yes | Results reported for all models across all assessment domains. | Results |
| Data availability | Yes | Data availability statement included. | End matter |
| Funding disclosure | Yes | Funding statement included. | End matter |
| Conflict of interest disclosure | Yes | Conflict of interest statement included. | End matter |
