# Supplementary material for: Performance of ChatGPT, Claude, and AMBOSS on the European Board of Urology In-Service Assessment and Alignment With the European Association of Urology 2025 Guidelines: Comparative Study
Source: JMIR Form Res. 2026 Sep 2;10:e100148. doi: 10.2196/100148 (PMC13583496; doi:10.2196/100148)
Supplement: Supplementary file 2 [file formative_v10i1e100148_app2.docx]

**Multimedia Appendix 2.** Non-obvious deductive, discriminative reasoning and clinical validity insight levels across AI models.

Percentages reflect the proportion of 200 questions rated as low, moderate, or high insight for ChatGPT 4.0, Claude, and AMBOSS.

| **AI model** | **Low, n (%)** | **Moderate, n (%)** | **High, n (%)** |
| --- | --- | --- | --- |
| **Non-obvious deductive** |  |  |  |
| ChatGPT 4.0 | 94 (47.0) | 97 (48.5) | 9 (4.5) |
| Claude | 96 (48.0) | 98 (49.0) | 6 (3.0) |
| AMBOSS | 120 (60.0) | 78 (39.0) | 2 (1.0) |
| **Discriminative reasoning** |  |  |  |
| ChatGPT 4.0 | 21 (10.5) | 120 (60.0) | 59 (29.5) |
| Claude | 20 (10.0) | 140 (70.0) | 40 (20.0) |
| AMBOSS | 123 (61.5) | 66 (33.0) | 11 (5.5) |
| **Clinical validity** |  |  |  |
| ChatGPT 4.0 | 14 (7.0) | 0 (0) | 186 (93.0) |
| Claude | 40 (20.0) | 0 (0) | 160 (80.0) |
| AMBOSS | 50 (25.0) | 0 (0) | 150 (75.0) |
